# Identification of a three-m6A related gene risk score model as a potential prognostic biomarker in clear cell renal cell carcinoma

**DOI:** 10.7717/peerj.8827

**Published:** 2020-03-18

**Authors:** Yiqiao Zhao, Zijia Tao, Xiaonan Chen

**Affiliations:** Department of Urology, Shengjing Hospital of China Medical University, Shenyang, Liaoning, China

**Keywords:** m6A, Risk score, Prognosis, ccRCC

## Abstract

**Background:**

Clear cell renal cell carcinoma (ccRCC) is one of the most prevalent malignancies worldwide, N6-methyladenosine (m6A) has been shown to play important roles in regulating gene expression and phenotypes in both health and disease. Here, our purpose is to construct a m6A-regulrator-based risk score (RS) for prediction of the prognosis of ccRCC.

**Methods:**

We used clinical and expression data of m6A related genes from The Cancer Genome Atlas (TCGA) dataset and the Least Absolute Shrinkage and Selection Operator (LASSO) Cox regression analysis to develop an RS to predict survival of patients with ccRCC, and analyzed correlations between RS and other clinical indicators such as age, grade and stage. Validation of this RS was then engaged in another cohort, E-MTAB-1980 from the ArrayExpress dataset. Finally, we used quantitative real-time PCR to analyze the expression profile of genes consists of the RS.

**Results:**

A three-gene RS including METTL3, METTL14 and HNRNPA2B1 which can predict overall survival (OS) of ccRCC patients from TCGA. After applying this RS into the validation cohort from Arrayexpress, we found that it successfully reproduced the result; furthermore, the results of PCR validation were in line with our analysis.

**Conclusion:**

To sum up, our study has identified an RS composed of m6A related genes that may predict the prognosis of ccRCC patients, which might be helpful for future therapeutic strategies. Our results call for further experimental studies for validations.

## Introduction

Renal cell carcinoma (RCC) is a frequent malignant tumor of the adult kidney (Siegel, Miller & Jemal, 2018), approximately 75% of RCC patients have the clear cell renal cell carcinoma subtype (ccRCC) (Cheville et al., 2003). The main and most effective treatment for ccRCC is radical nephrectomy. However, 30% of patients experience recurrence and progression which significantly reduces the overall survival (OS) of patients (Lane & Kattan, 2008). Additionally, ccRCC is generally resistant to radiotherapy and chemotherapy (Rini, Campbell & Escudier, 2009). Therefore, a better comprehension of the molecular mechanisms underlying ccRCC is helpful to develop new therapies for this disease.

In recent years, epitranscriptomics (post-translational RNA modifications) have been shown to play fundamental roles in many biological and disease processes (Davalos, Blanco & Esteller, 2018), among which, methylation of N6-methyladenosine (m6A) is the most abundant form of messenger RNA (mRNA) modification in eukaryotes (Liu & Pan, 2016). It is widely acknowledged that regulations of m6A are accomplished by a diversity of proteins which were grouped into “writers”, “readers” and “erasers” (Liu & Pan, 2016; Zhang et al., 2018; Schaefer, Kapoor & Jantsch, 2017; Meyer & Jaffrey, 2017; Jacob, Zander & Gutschner, 2017; Frye et al., 2016). The linkages of m6A and various tumors have been explored by plenty of researchers recently, including testicular germ cell tumors (Lobo et al., 2019), pancreatic cancers (Taketo et al., 2018), myeloid leukemia (Barbieri et al., 2017) and colorectal cancers (Nishizawa et al., 2018).

Moreover, several studies focused on m6A regulators in patients with ccRCC in recent years, for example, Zhou et al. (2019) have reported the relationship between mutation and copy number variation (CNV) of m6A regulatory genes with ccRCC. They found that the deletion of m6A “writer” genes was an independent prognostic factor for ccRCC patients, and copy number gain of m6A “eraser” genes could enhance the effect. In addition, ccRCC patients with any CNVs of m6A regulatory genes had worse OS and DFS than those with diploid genes (Zhou et al., 2019). Besides, Li et al. (2019) have revealed that m6A reader IGF2BP3 maybe a potential oncogene for ccRCC and other 32 cancers through a pan-cancer analysis the study was mainly to investigate the general rule of m6A regulators across 33 cancer types. Nevertheless, few studies construct a risk score (RS) based on mRNA expression of m6A regulatory genes to predict the prognosis of ccRCC patients. In general, the prediction efficiency of RS model is better than that of single gene model.

In the current study, we aimed to explore the relationships between expression levels of m6A writers (Wilms Tumor 1-Associating Protein (WTAP) Vir like m6A methyltransferase associated (KIAA1429/VIRMA), methyltransferase like (METTL) 3, 4, 14 and 16, RNA binding motif protein 15 (RBM15), RBM15B). Its readers (Heterogeneous nuclear ribonucleoprotein C and A2–B1 (HNRNPC and HNRNPA2B1) YTH domain family proteins (YTHDF) 1, 2 and 3, YTH domain-containing proteins (YTHDC) 1 and 2) and its erasers, such as α-ketoglutarate dependent dioxygenase 5 (ALKBH5) and fat mass and obesity related (FTO) (Lobo et al., 2018). Expression and clinical data from The Cancer Genome Atlas (TCGA) was applied to construct an RS that can be helpful in prognostic assessment of ccRCC; we furthermore downloaded data from ArrayExpress to validate the rationality of the RS.

## Methods and materials

### Collection of primary data

The TCGA-KIRC mRNA FPKM transcriptome profiling data (with 539 tumor samples and 72 normal samples) and associated clinical information (*N* = 491) were downloaded from TCGA (http://cancergenome.nih.gov/), we then extracted the expression data of these seventeen m6A genes for following operations.

### Consensus clustering of m6A genes

Identification of distinct clusters of ccRCC patients using consensus clustering was performed primarily, by which we decided to divide patients into *k* (*k* = 2–9) clusters according to the expression of m6A genes, moreover, we did survival analyses of these *k* clusters to see whether the clustering is reasonable.

### Data processing and DEGs analysis

DEGs were filtered by comparing the expression level of ccRCC samples and normal samples in TCGA dataset utilizing the wilcox test in Limma package in R software with the cutoff criteria of *p*-value < 0.05. A violin plot was depicted to clearly display the differential expression of m6A genes.

### Selection of potential prognostic-related genes

We did univariate cox analysis of all DEGs by survival package in R software. The DEGs with a *p*-value less than 0.05 were considered as prognostic-associated genes and selected as candidate genes for further procedure.

### Construction of RS

In order to gain better prediction of m6A genes and ccRCC, we compared three analysis models (Least Absolute Shrinkage and Selection Operator (LASSO), Ridge and Elastic Net analysis) to construct better RS. Finally, we implemented LASSO COX regression analysis to candidate genes from TCGA (which was used as a training set) utilizing the survival and glmnet packages in R. Finally, genes and their coefficients were determined. The RS was calculated by the formula:

$${\rm RS}=\sum^{n}_{i=1} {\rm coefi}\times{x_i}$$

In which the coefi is the coefficient, and *x_i_* is the expression value of each selected gene. This formula was used to calculate a RS for each patient in both the training (TCGA) dataset.

### OS analysis of RS

To investigate the relationship between the RS and 5 year OS, we used survival package in R to map a Kaplan–Meier plot (K–M plot) by TCGA data, patients were divided into high and low risk groups by the median value of RS, *p* < 0.05 was applied as cut-off standard. In addition, we plotted a ROC curve by survivalROC package, moreover, the AUC, was calculated to predict the accuracy of the survival analysis.

### Cox regression analysis of RS

We ran univariate and multivariate cox regression analyses by survival and forestplot packages to inspect the independency of RS as a prognostic factor. Except for RS, we analyzed the impact of age (which was classified into two groups: >60 and ≤60), gender, T stage, M stage, grade and stage. All the ambiguous value are excluded, including Mx, Tx N/A and unknown (we deleted all the N stage data about TCGA patients because the large amount of Nx). Each factor with a *p* value < 0.05 in univariate analysis was then analyzed by multivariate cox regression, those with a *p* value < 0.05 and HR > 1 were viewed as independent prognostic factors.

### Validation of the RS

For the purpose of RS validation, we downloaded another dataset, E-MTAB-1980 (*N* = 101) from ArrayExpress (https://www.ebi.ac.uk/arrayexpress/). However, the expression data from this validation set was log2 processed, thereby, we reversely normalized the data so that it is in consistent with that of TCGA. The RS formula was then applied to each sample in validation set, likewise, the patients were separated to high and low risk groups in term of median RS value. Eventually, a K–M plot and an ROC curve were constructed for verification.

### Validation by quantitative real-time PCR from clinical specimens of ccRCC

To further validate our finding from TCGA and ArrayExpress database, we performed quantitative real-time PCR (qRT-PCR) to detect expression of HNRNPA2B1, METTL3 and METTL14 in 16 pairs of ccRCC samples and matched adjacent normal kidney tissues (*n* = 32). All patients received radical nephrectomy from January 2017 to March 2018 in Shengjing Hospital of China Medical University and were pathologically diagnosed as ccRCC. This study was approved by Ethics Committee of Shengjing Hospital (No. 2019PS050J), all patients provided written scientific ethics consents.

RNA was extracted from frozen samples using TRIzol Reagent (Invitrogen, Carlsbad, CA, USA) following the manufacturer’s instructions. RNA purity and concentrations were identified, then reverse transcription and qRT-PCR were performed. Detailed procedures had been described in our previous paper (Chen et al., 2016). U6 acted as internal control to normalize the results. The relative quantification equation (RQ = 2^−ΔΔCt^) was used to calculate the relative expression of RNA.

## Results

### Clustering of m6A genes

**Figure 1:**
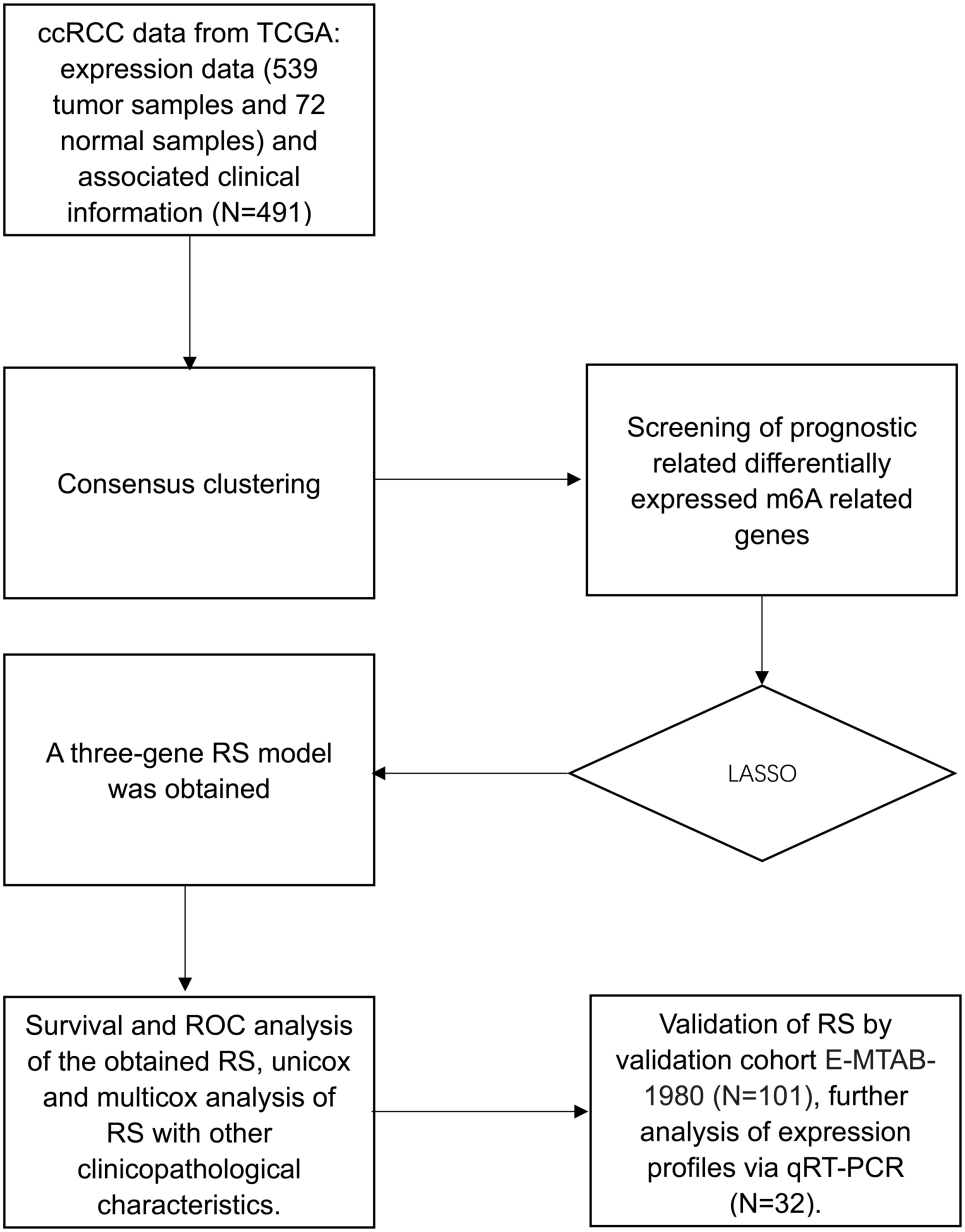
Flow chart. Flow chart of data preparation, processing analysis and validation in this study.

**Figure 2:**
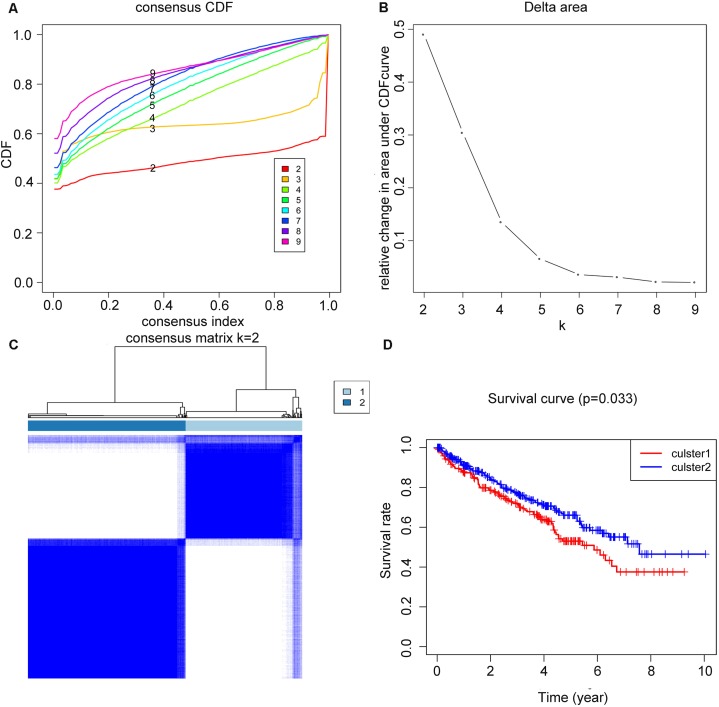
Clustering of TCGA–KIRC patients and overall survival (OS) of the two clusters. (A) Consensus clustering cumulative distributive function (CDF) for *k* = 2–9, the lines were labeled in numerical order from the bottom to the top. (B) Relative change in area under CDF curve for *k* = 2–9. (C) Patients of TCGA–KIRC was divided into two clusters when *k* = 2. (D) Kaplan–Meier overall survival (OS) curve for ccRCC patients between the two clusters.

The overall workflow of the process that we used to develop and validate the RS to predict prognostic outcomes was depicted in Fig. 1. Based on the expression similarity of m6A genes, *k* = 2 seemed to be an adequate selection with clustering stability increasing from *k* = 2 to 9 in the TCGA datasets, which we named as cluster1 and cluster2 (Figs. 2A–2C). Significant difference of OS was noticed between these two clusters (*p* = 0.033) (Fig. 2D), we believed that the results may reveal a potential correlation of m6A genes and ccRCC patients, which led to the following exploration.

### DEGs and prognostic-related genes screening

**Figure 3:**
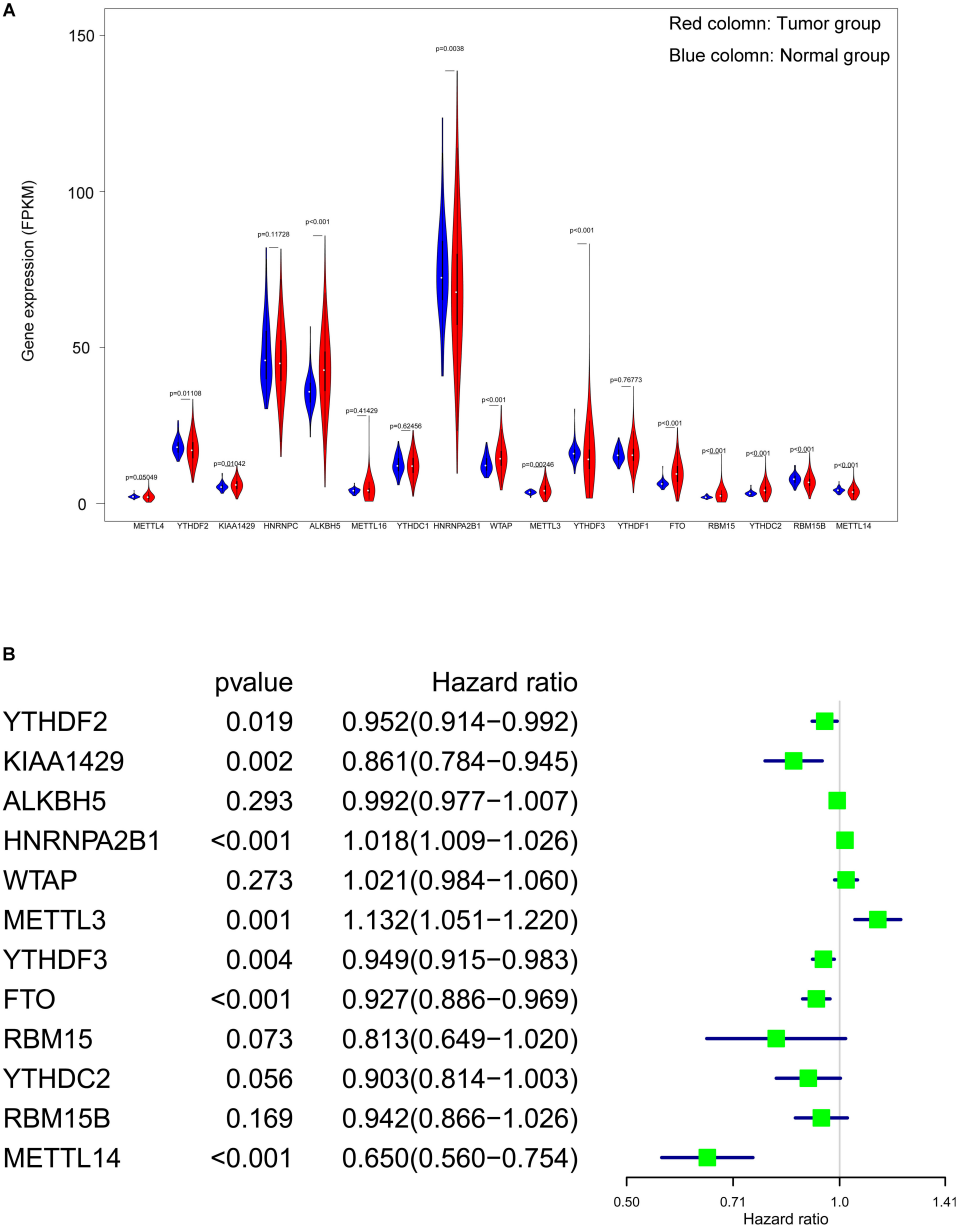
Screening of differentially expressed and prognostic related m6A related genes. (A) Violin plot of selection of differentially expressed m6A related genes (*p* < 0.05), the tumor and normal samples were represented as red and blue group respectively. (B) Identification of m6A related genes that has correlation with the OS.

From 17 m6A genes, a total of 12 DEGs with a *p* value < 0.05 were identified. Including YTHDF2, KIAA1429, ALKBH5, HNRNPA2B1, WTAP, METTL3, YTHDF3, FTO, RBM15, YTHDC2, RBM15B and METTL14. The expression level of all 17 genes were illustrated in Fig. 3A. Additionally, univariate cox regression analysis of the DEGs was operated, seven genes (YTHDF2, KIAA1429, HNRNPA2B1, YTHDF3, METTL3, FTO and METTL14) were considered as prognostic-related genes, the results was shown in Fig. 3B.

### Formula and OS analysis of the RS

**Figure 4:**
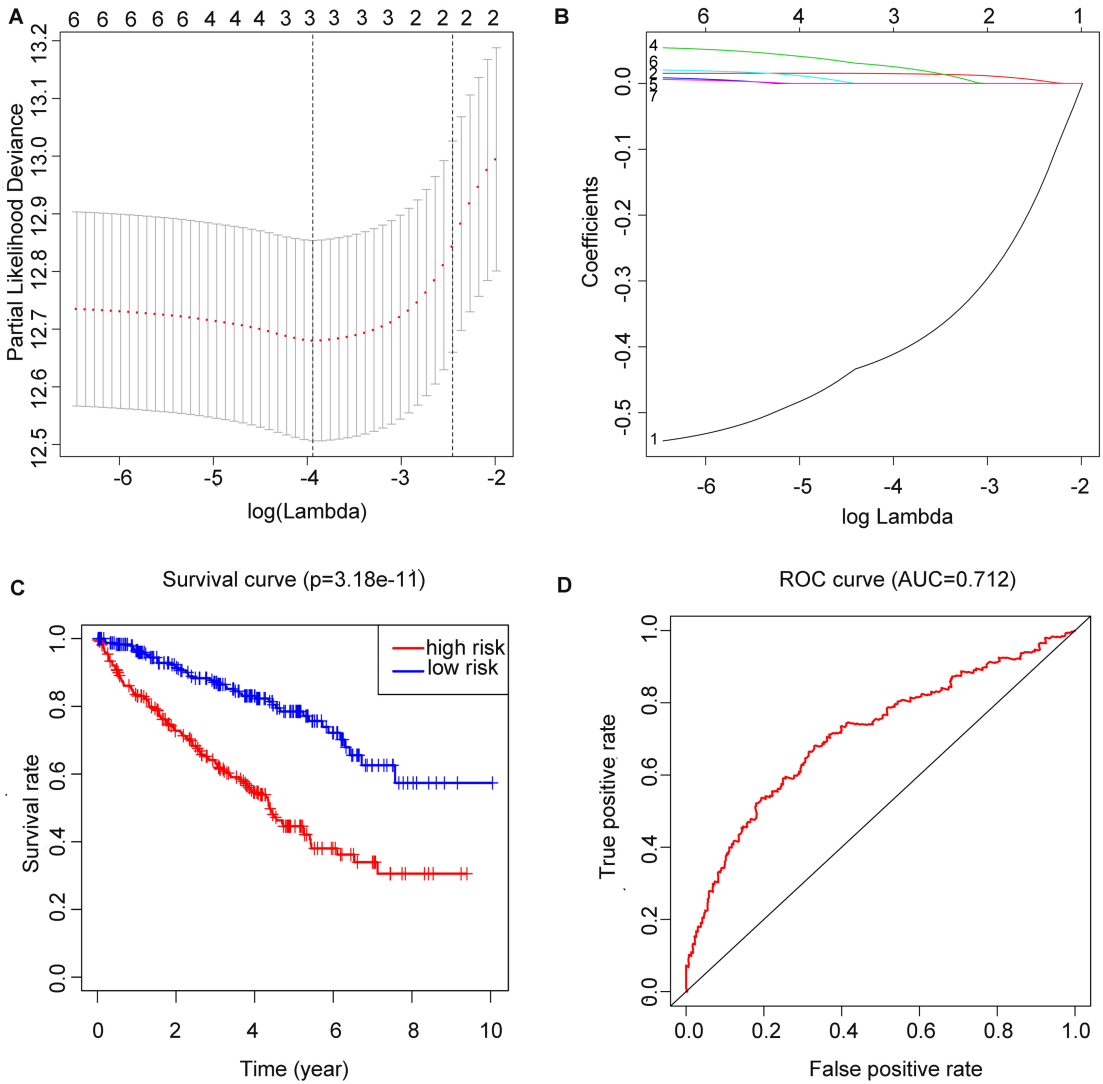
Construction of m6A related risk score model and assessment of its prognostic value. (A and B) Least absolute shrinkage and selection operator (LASSO) Cox regression algorithm to seven prognostic which we used to construct the risk score (RS) model, the labels in (B) were arranged in the order of the lines from top to bottom. (C) ****Kaplan–Meier plots of the OS of patients from TCGA to KIRC. (D) ROC curve which was used for evaluation of the prediction efficiency of the RS model.

After running the LASSO COX regression analysis of the seven genes, three genes (METTL14, HNRNPA2B1 and METTL3) were filtered and selected to build the prediction model, as is demonstrated in (Figs. 4A and 4B). The total RS was imputed as follows: (−0.413491830046862 × expression level of *METTL14*) + (0.0149601455134957 × expression level of *HNRNPA2B1*) + (0.0260335348056223 × expression level of *METTL3*). The 5-year OS and ROC curve were presented in Figs. 4C and 4D while we found significant differences between the high and low risk groups (*p* = 3.18E−11), the time-dependent AUC further proved the accuracy of the result (AUC = 0.712).

### RS and other clinicopathological characteristics

**Figure 5:**
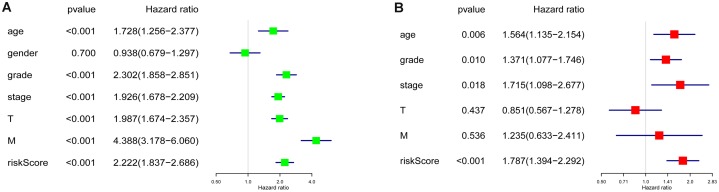
Cox regression analyses of the RS and clinicopathological parameters to select factors significantly associated with the OS. (A) Univariate Cox analysis. (B) Multivariate Cox analysis.

Age, grade, stage, T stage, M stage and RS showed statistical significance after the univariate analysis (all *p* value < 0.001), which is illustrated in Fig. 5A, these six factors were then involved in multivariate analysis when we observed age (*p* = 0.006), grade (*p* = 0.010), stage (*p* = 0.018) and RS (*p* < 0.001) remaining to be significant (Fig. 5B). Besides, all four factors were with an HR that is bigger than one.

### Performance of RS in validation set

**Figure 6:**
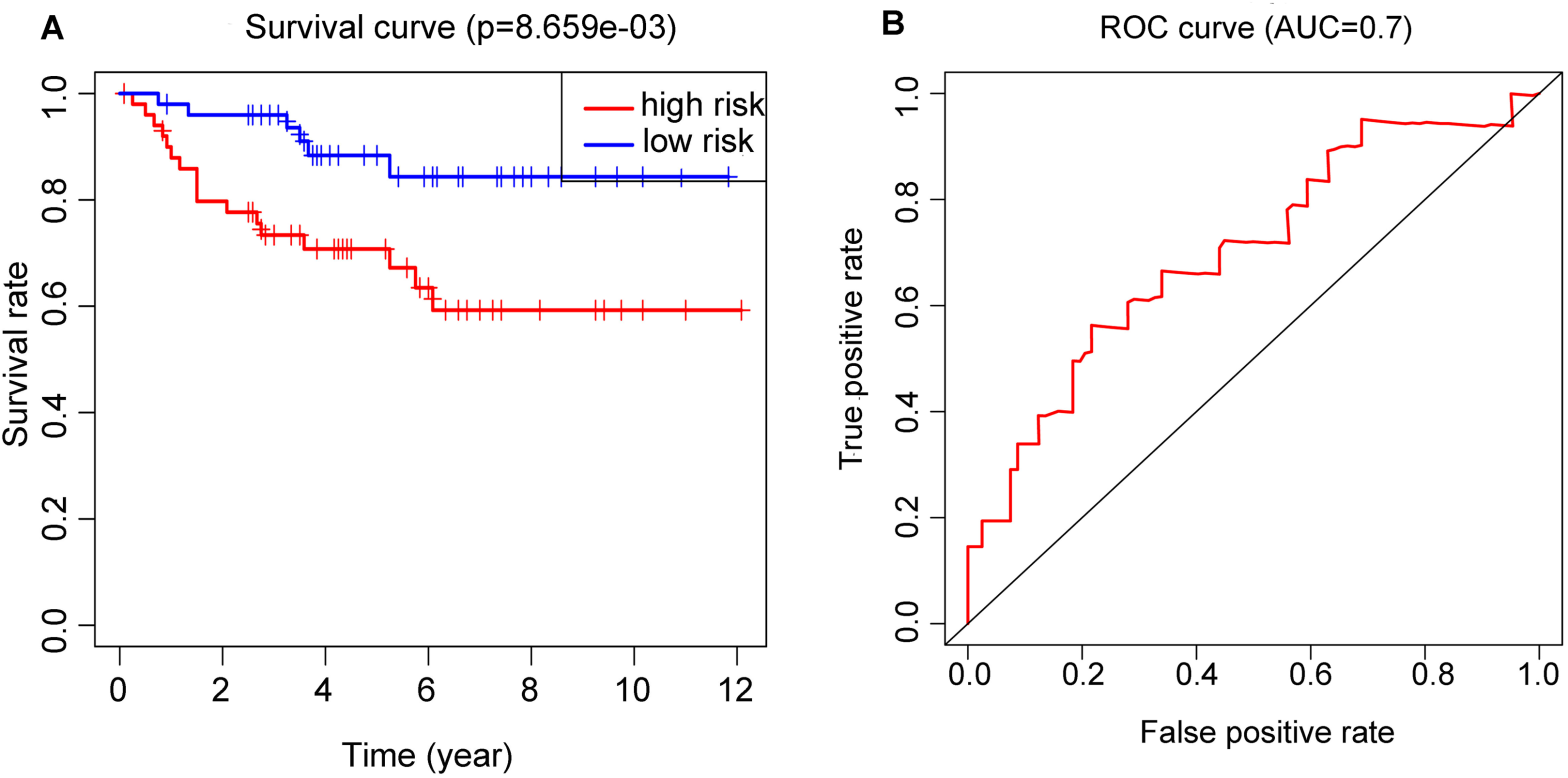
Application of the RS model in the validation cohort. (A) Kaplan–Meier plots of the OS validation cohort. (B) ROC curve which was used for evaluation of the prediction efficiency of the RS model in the validation cohort.

OS and ROC curve constructed by E-MTAB-1980 dataset (Figs. 6A and 6B) displayed similar results as that in the training set. The *p* value = 8.659E−03 and AUC = 0.7 all meet the standard of significance. The result confirmed that the RS attained in our study may be a potential prognostic biomarker.

### Comparison of LASSO, ridge and elastic net model

In addition to LASSO COX regression analysis, Ridge and Elastic Net analysis were also applied two different RS were obtained. We then did survival and cox analysis via these two RSs. We got meaningful results by all three models in training cohort (Figs. S1–S3). Nevertheless, the Ridge model showed no significance for prognosis in the validation cohort (Fig. S4C). Despite Elastic Net model generated smaller *p* value to LASSO model in the training cohort (Figs. S1A and S1B), but got higher *p* value than LASSO model in the validation cohort (Figs. S4A and S4B). Moreover, its AUC of the ROC curve in the validation dataset was 0.686, which did not meet the cut-off standard that AUC ≥ 0.7(Fig. S5). Therefore, we finally chose lasso model to construct RS.

### Unicox survival analysis of m6A genes and RS in validation cohort

Forest plot of m6A related genes and RS was mapped (Fig. S6), although all RS related genes showed weak significance in the validation cohort (METTL14, HR = 0.986 (0.9775–0.9945); METTL3, HR = 0.9993 (0.9980–1.0007); HNRNPA2B1, HR = 1.0006 (1.0004–1.0008)), the RS we obtained still had comparative advantage over other genes (RS *p* < 0.001, HR = 1.0236 (1.0118–1.0355)).

### Validation based on qRT-PCR

**Figure 7:**
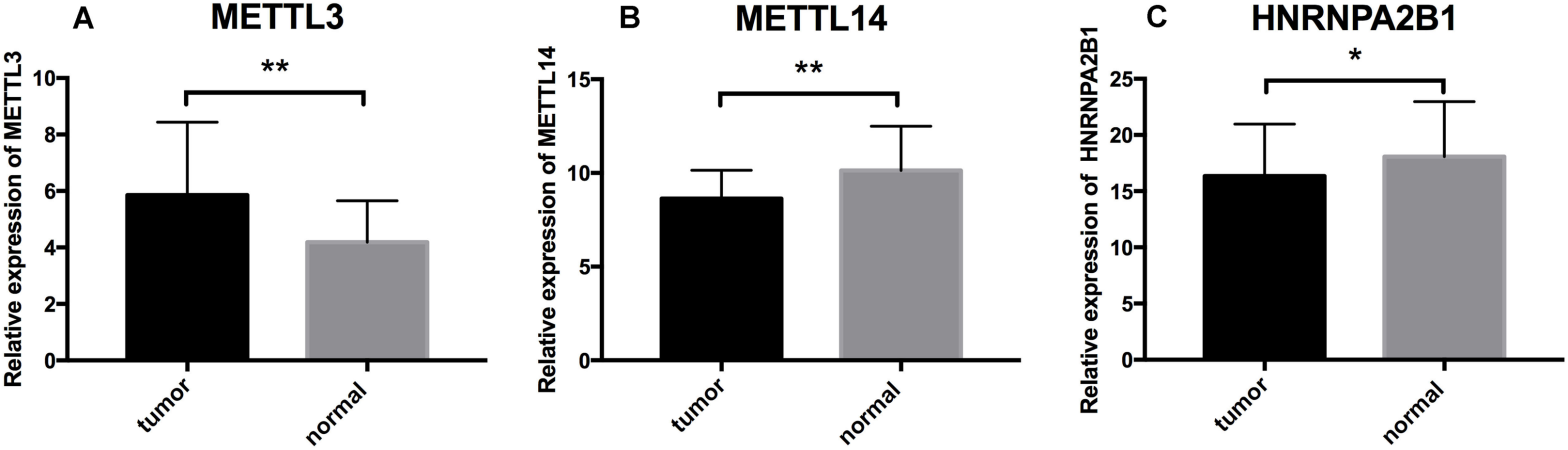
Further analysis of the expression profiles of RS component genes through qRT-PCR. (A) Expression profiles of METTL3. (B) Expression profiles of METTL14. (C) ****Expression profiles of HNRNPA2B1. * *p* < 0.05, ** *p* < 0.01, *p* < 0.05 was taken as a cut-off standard.

To further validate the expression pattern of the three differentially expressed genes (METTL14, HNRNPA2B1 and METTL3) from our RS model. The qRT-PCR was performed on 16 paired ccRCC samples and matched adjacent normal kidney tissues. The expression level of METTL3 was significantly higher in ccRCC specimens than those in normal tissues (*p* = 0.0095) (Fig. 7A). Inversely, the expression of METTL14 and HNRNPA2B1 were relatively lower in tumor tissues (*p* = 0.0016, *p* = 0.0163 respectively) (Figs. 7B and 7C). These results were consistent with our finding based on TCGA database.

## Discussion

In the past few years, it is not uncommon to see researches about single prognostic biomarker in ccRCC. However, due to the heterogenous nature of kidney cancer (Sankin et al., 2014), single biomarker lacks ample credibility to predict patients’ outcomes, thereby, an RS that is composed of more than one biomarker is necessary. There were a few former articles about ccRCC and RS models which were based on gene expression (Zeng et al., 2019; Li et al., 2018), lncRNA expression (Liu et al., 2018; Zhang et al., 2019) or miRNA expression (Luo et al., 2019; Fritz et al., 2014), because of the tremendous amount of genes and various RNAs the models of these studies were diverse. Currently, as the importance of m6A cut a striking figure, despite the fact that the first report of m6A was as early in 1974 (Desrosiers, Friderici & Rottman, 1974), it was not until recent days that the mechanism of the m6A was uncovered (Roundtree et al., 2017). Moreover, plenty of researches has disclosed the associations of m6A modification in former mentioned cancerous diseases (Lobo et al., 2019; Taketo et al., 2018; Barbieri et al., 2017; Nishizawa et al., 2018) or non-cancerous diseases such as type 2 diabetes mellitus (Bohnsack & Sloan, 2018), and infertility (Ding et al., 2018), thus, we decided to build an RS model found on m6A genes.

In our study, by using TCGA dataset, 12 DEGs were first screened out of 17 m6A related genes, within which we further found seven genes that showed potential significance in prognostic value. Through the LASSO Cox regression model, a three-gene (METTL14, HNRNPA2B1 and METTL3) signature was finally obtained. K–M plot and ROC curve both showed the reasonability of our RS model. Afterwards, the model was tested in the validation set downloaded from ArrayExpress, hopefully, the result was in accordance with the training cohort, which justified the RS model. We then used clinical tissues to further analyze the expression profiles of the above three genes in ccRCC patients, and found that METTL14 and HNRNPA2B1 showed higher expressions in normal samples than in tumor tissues, while METTL3 expression in tumor tissues were higher than that of normal tissues, which was in accordance with our analysis. In spite of the fact that all m6A regulatory genes showed weak significance in the validation cohort from unicox analysis, the RS we obtained still had comparative advantage over single genes to predict survival of ccRCC patients. However, we obtained meaningful K–M plot and ROC in validation cohort based on our RS model. We presumed that the reason is due to there are fewer patients, shorter mean follow-up time, and lower mortality in validation cohort than that in TCGA database. So we will try to validate our RS model in new qualified dataset in the future.

All three genes that composed the RS were studied before, there was a study uncovering the promotion function by METTL3 on post-transcriptional regulation of gene expression in acute myeloid leukemia. Barbieri et al. (2017), down-regulation of METTL14 was reported to act as an adverse prognosis factor for recurrence-free survival of hepatocellular carcinoma (Ma et al., 2017), in addition, HNRNPA2B1 was found to promote the development of epithelial–mesenchymal transition of pancreatic cancer by down-regulating E-cadherin and up-regulating N-cadherin and vimentin (Dai et al., 2017). Furthermore, METTL3 and METTL14 were reported to be correlated with ccRCC as was mentioned above (Zhou et al., 2019), in this research, Zhou et al. (2019) focused on the mutations and copy number variations (CNVs) of m6A related genes and found that these two m6A writers may form a complex that function in certain pathways which are involved in m6A regulation in ccRCC cancer cells, in our study, utilizing expression analysis and Lasso regression analysis, we obtained an RS model consists of the above two writers with an m6A reader HNRNPA2B1 that presented relatively strong statistical power in both training and validation cohort, in addition, Woodcock et al. (2019) found that human METTL3–METTL14 complex is active in vitro as a DNA adenine-N6 MTase, Schöller et al. (2018) delineated the overall architecture of METTL3/METTL14–WTAP complex. Moreover, intriguingly, the unicox survival analysis of the validation cohort disclosed the extraordinary significance of RBM15 comparing to other differentially expressed genes, RBM15 has also been reported to support interactions between METTL3/14 complex and substrate RNA (Patil et al., 2016). All these articles indicated that m6A regulators interact with each other to function in human cells, so there is a possibility that HNRNPA2B1 interact with METTL3/14 complex so that they function in ccRCC cells. However, the specific molecular mechanism between these three genes still requires further experiments and studies for exploration and validation.

Limitations certainly exists in this study. First, races and gender of ccRCC patients, stages and aggressiveness of ccRCC were not distinguished, which requires deeper exploration. Second, although validation cohort is constructed, it is far from enough, further verifications are always essential. Additionally, despite the fact that validation by TCGA data of our results were performed, functional experiments in vitro and vivo have not been conducted.

## Conclusion

In conclusion, we built up a three-m6A methylation regulators-based RS of METTL3, METTL14 and HNRNPA2B1 that may become potential biomarker for prognosis prediction of clear cell renal cell carcinoma patients, our results call for further experimental studies for validations.

## Supplemental Information

10.7717/peerj.8827/supp-1 Supplemental Information 1 K–M plots of LASSO, Elastic Net and Ridge models in training cohort. A. Overall survival of LASSO model in the training cohort B: Overall survival of Elastic Net model in the training cohort C. Overall survival of Ridge model in the training cohort

10.7717/peerj.8827/supp-2 Supplemental Information 2 ROC curve of LASSO, Elastic Net and Ridge models in training cohort. A. Time-dependent ROC curve for accuracy of the LASSO model in training cohort. B: Time-dependent ROC curve for accuracy of the Elastic Net model in training cohort. C. Time-dependent ROC curve for accuracy of the Ridge model in training cohort.

10.7717/peerj.8827/supp-3 Supplemental Information 3 Cox regression analyses of the RS and clinicopathological parameters to select factors significantly associated with the OS. A and B: Univariate Cox analysis and Multivariate Cox analysis by LASSO model, C and D: Univariate Cox analysis and Multivariate Cox analysis by Elastic Net model, E and F: Univariate Cox analysis and Multivariate Cox analysis by Ridge model.

10.7717/peerj.8827/supp-4 Supplemental Information 4 K-M plots of LASSO, Elastic Net and Ridge models in validation cohort. A. Overall survival of LASSO model in the validation cohort B: Overall survival of Elastic Net model in the validation cohort C. Overall survival of Ridge model in the validation cohort

10.7717/peerj.8827/supp-5 Supplemental Information 5 ROC curve of LASSO and Elastic Net models in validation cohort. A. Time-dependent ROC curve for accuracy of the LASSO model in validation cohort. B: Time-dependent ROC curve for accuracy of the Elastic Net model in validation cohort.

10.7717/peerj.8827/supp-6 Supplemental Information 6 Forest plot of unicox regression analysis based on clinical data from validation cohort.

10.7717/peerj.8827/supp-7 Supplemental Information 7 Expression of HNRNPA2B1 in 16 pairs of ccRCC samples and matched adjacent normal kidney tissues (n=32).

10.7717/peerj.8827/supp-8 Supplemental Information 8 Expression of METTL3 in 16 pairs of ccRCC samples and matched adjacent normal kidney tissues (n=32).

10.7717/peerj.8827/supp-9 Supplemental Information 9 Expression of METTL14 in 16 pairs of ccRCC samples and matched adjacent normal kidney tissues (n=32).

## Additional Information and Declarations

### Data Availability

The following information was supplied regarding data availability: The RNA-seq transcriptome data and corresponding clinicopathological information of the training cohort were obtained from TCGA: TCGA-KIRC: cases.project.project_id; HTSeq - FPKM: files.analysis.workflow_typ; Gene Expression Quantification: files.data_category; transcriptome profiling: files.data_type; TCGA-KIRC: cases.project.project_id; clinical: files.data_category; bcr xml: files.data_format. For the RNA-seq data, Fragments Per Kilobase of transcript per Million mapped reads (FPKM) values were obtained for the TCGA-KIRC samples. The validation cohort data is also available at ArrayExpress: E-MTAB-1980. The raw data is also available in the Supplemental Files. The raw data includes validation by qRT-PCR of expression of HNRNPA2B1, METTL3 and METTL14 in 16 pairs of ccRCC samples and matched adjacent normal kidney tissues (*n* = 32).
