# Supplementary figures and images for: Identification of a three-m6A related gene risk score model as a potential prognostic biomarker in clear cell renal cell carcinoma
Source: PeerJ. 2020 Mar 18;8:e8827. doi: 10.7717/peerj.8827 (PMC7085294; doi:10.7717/peerj.8827)

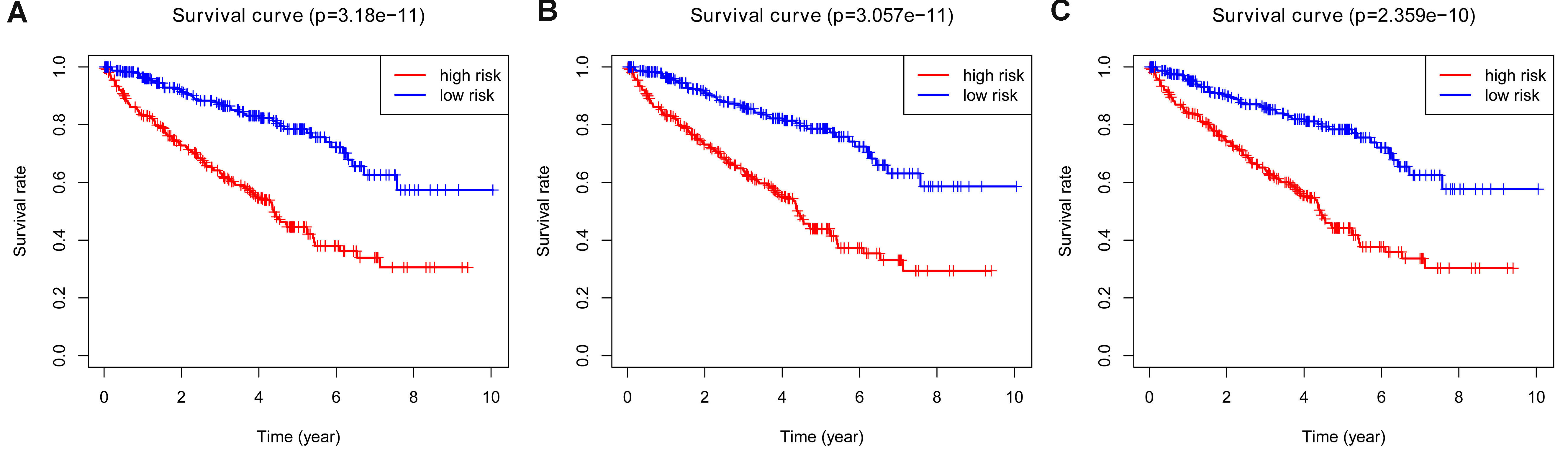

Supplement: Supplemental Information 1 — A. Overall survival of LASSO model in the training cohort B: Overall survival of Elastic Net model in the training cohort C. Overall survival of Ridge model in the training cohort [file peerj-08-8827-s001.png]

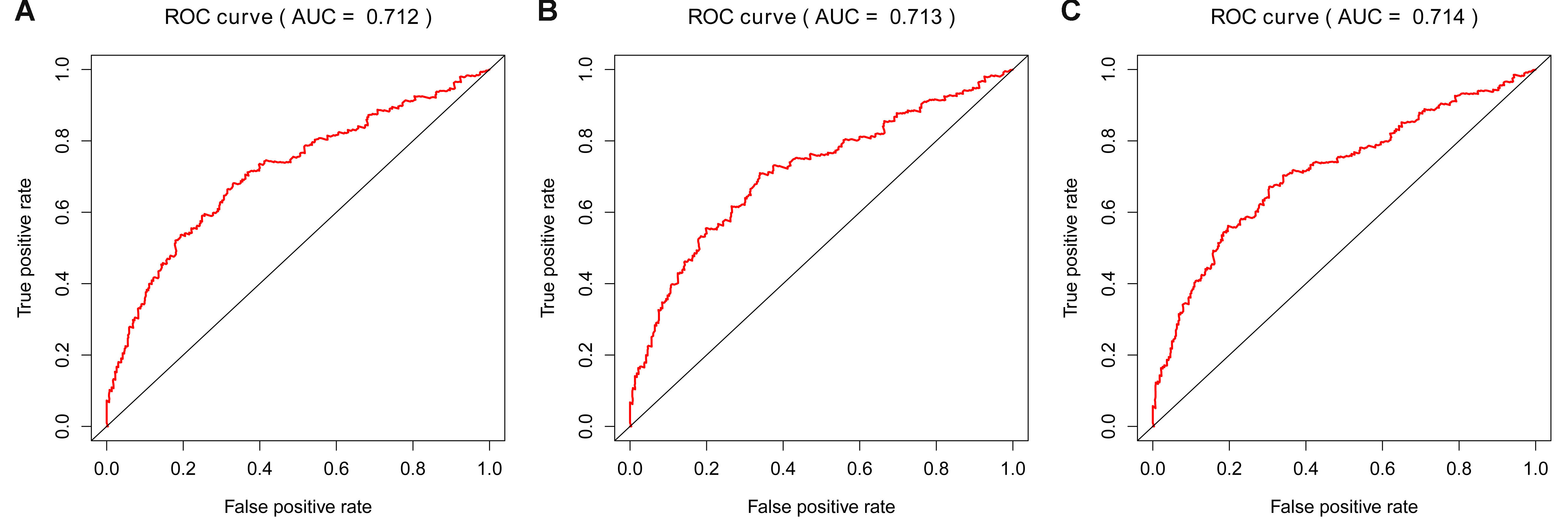

Supplement: Supplemental Information 2 — A. Time-dependent ROC curve for accuracy of the LASSO model in training cohort. B: Time-dependent ROC curve for accuracy of the Elastic Net model in training cohort. C. Time-dependent ROC curve for accuracy of the Ridge model in training cohort. [file peerj-08-8827-s002.png]

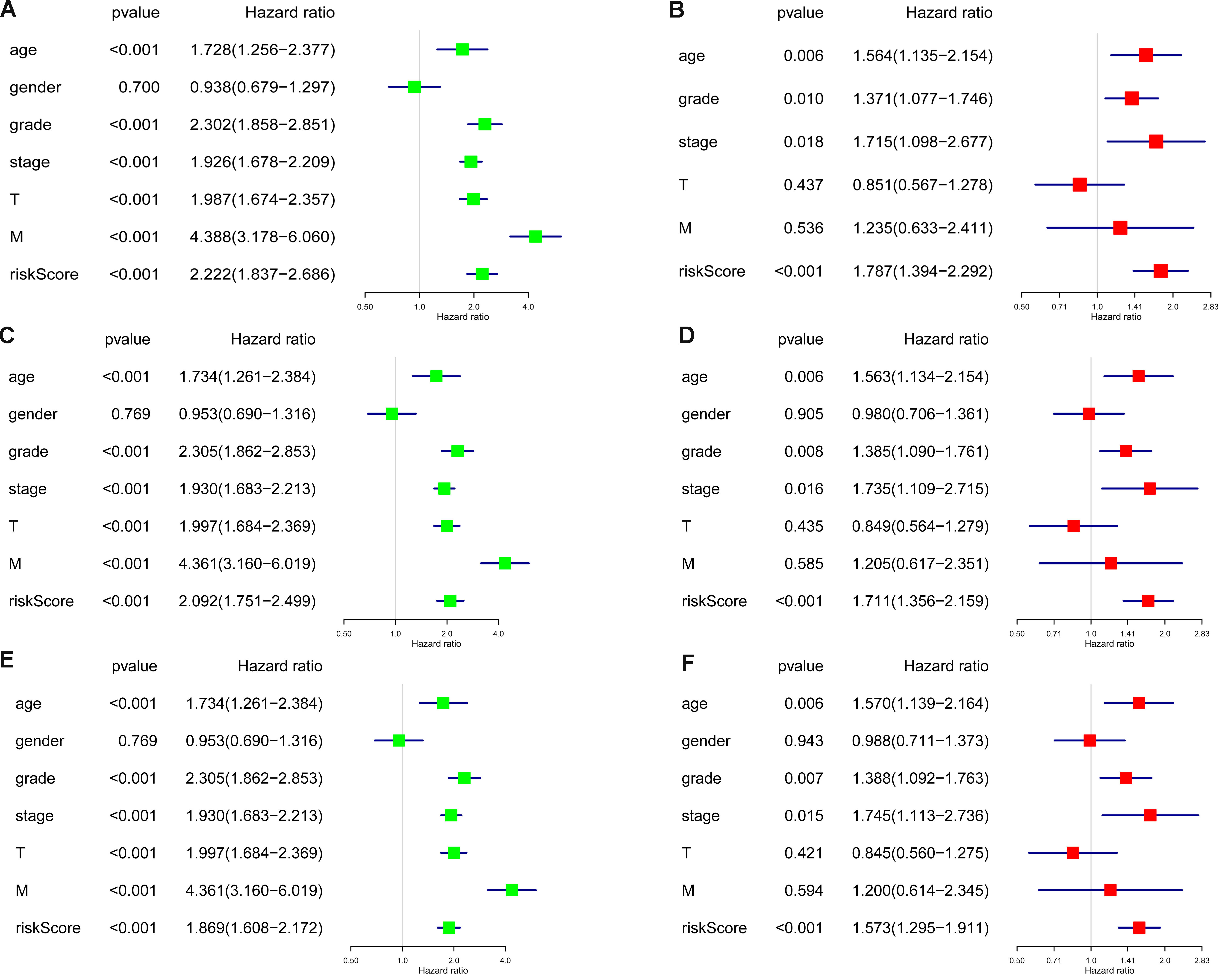

Supplement: Supplemental Information 3 — A and B: Univariate Cox analysis and Multivariate Cox analysis by LASSO model, C and D: Univariate Cox analysis and Multivariate Cox analysis by Elastic Net model, E and F: Univariate Cox analysis and Multivariate Cox analysis by Ridge model. [file peerj-08-8827-s003.png]

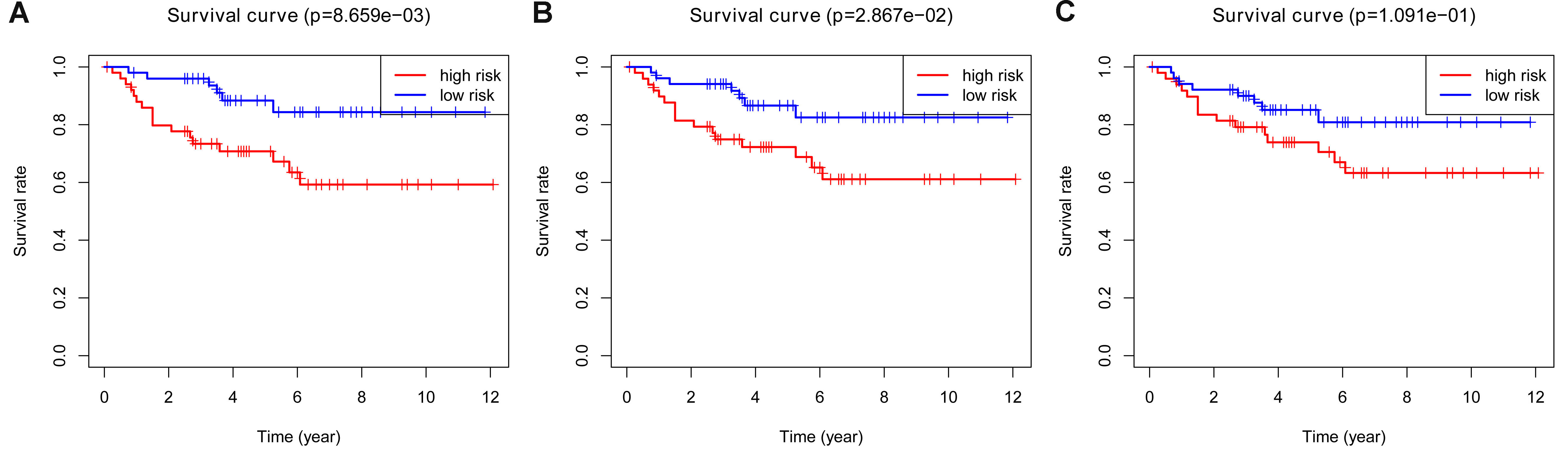

Supplement: Supplemental Information 4 — A. Overall survival of LASSO model in the validation cohort B: Overall survival of Elastic Net model in the validation cohort C. Overall survival of Ridge model in the validation cohort [file peerj-08-8827-s004.png]

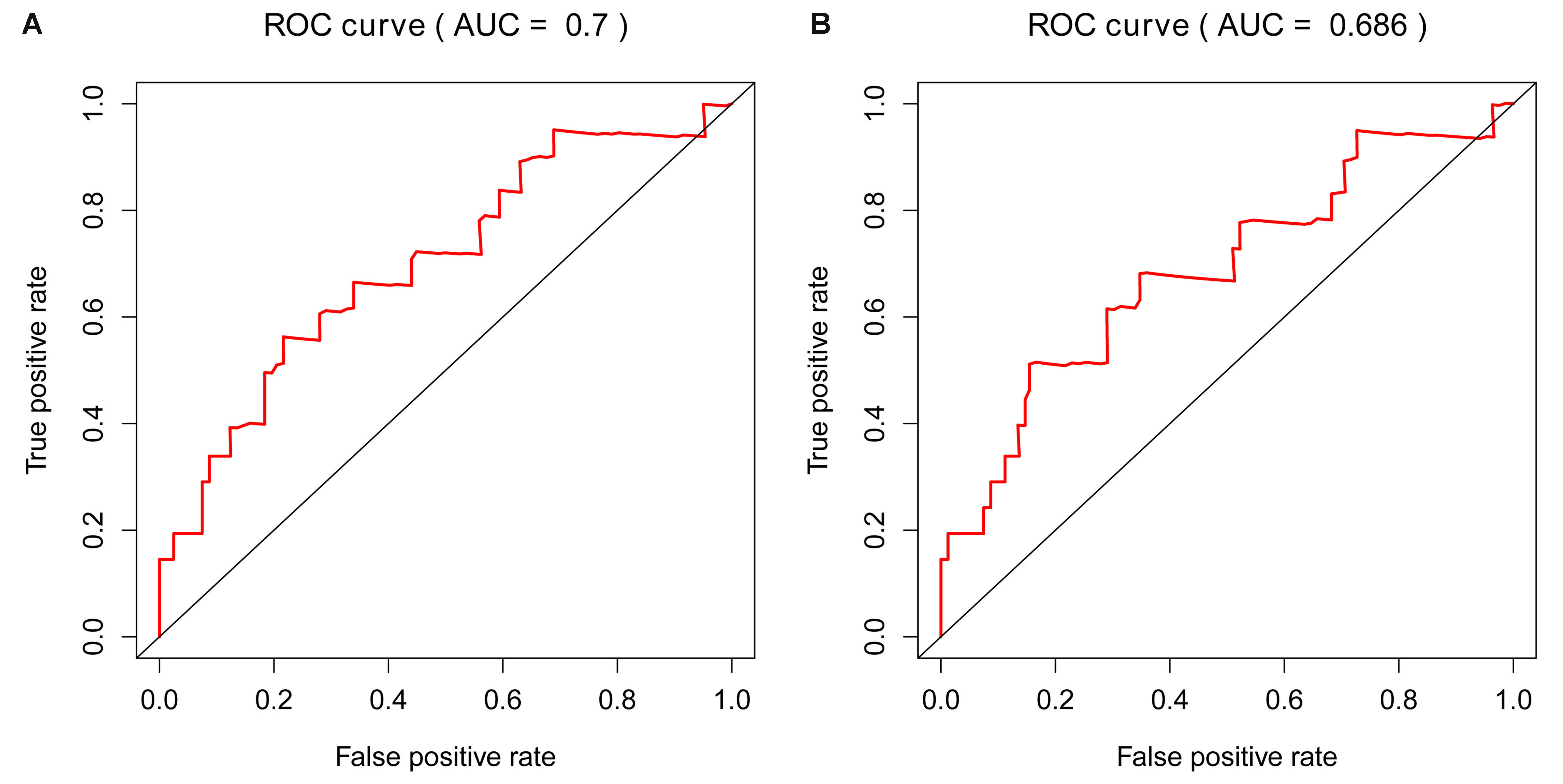

Supplement: Supplemental Information 5 — A. Time-dependent ROC curve for accuracy of the LASSO model in validation cohort. B: Time-dependent ROC curve for accuracy of the Elastic Net model in validation cohort. [file peerj-08-8827-s005.png]

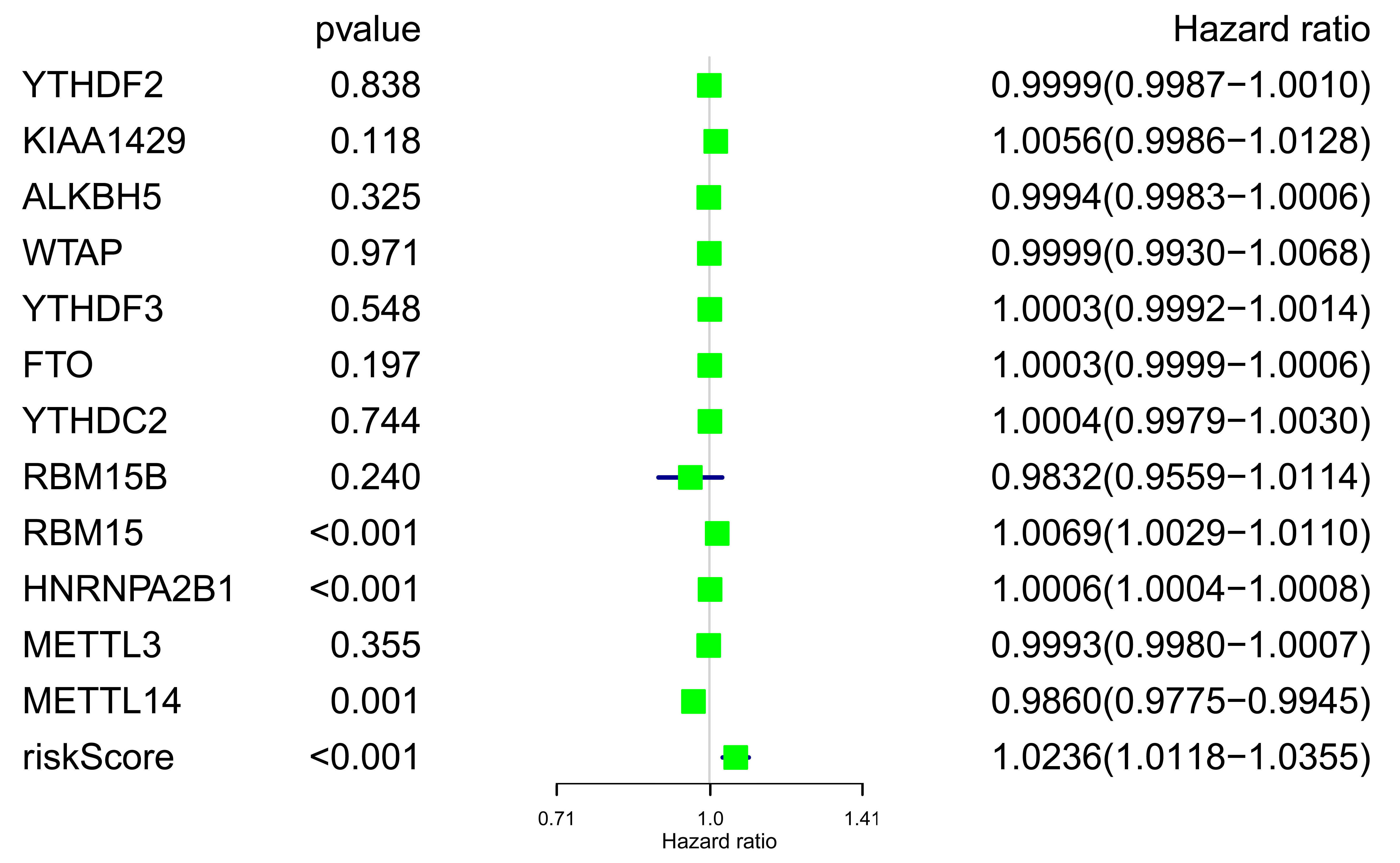

Supplement: Supplemental Information 6 [file peerj-08-8827-s006.png]
